# The association between gingivitis and oral spirochetes in young cats and dogs

**DOI:** 10.1371/journal.pone.0281126

**Published:** 2023-01-27

**Authors:** Seiya Yamaki, Masato Tachibana, Hisae Hachimura, Masao Ogawa, Shinya Kanegae, Hirokazu Amimoto, Takashi Shimizu, Kenta Watanabe, Masahisa Watarai, Akiteru Amimoto

**Affiliations:** 1 Amica Pet Clinic, Yamaguchi, Japan; 2 Joint Graduate School of Veterinary Medicine, Yamaguchi University, Yamaguchi, Japan; 3 Organization for Research Initiatives, Yamaguchi University, Yamaguchi, Japan; 4 Joint Faculty of Veterinary Medicine, Laboratory of Veterinary Public Health, Yamaguchi University, Yamaguchi, Japan; Universidade de Trás-os-Montes e Alto Douro: Universidade de Tras-os-Montes e Alto Douro, PORTUGAL

## Abstract

Although gingivitis frequently occurs in young cats, spirochetes are often found in the early stages of periodontal disease. This study was conducted to determine the association between gingivitis and oral spirochetes in young cats and dogs. The degree of gingivitis was evaluated in a total of 68 cats and 31 dogs under one year of age, and plaques were collected from each carnassial. To detect spirochetes or *Porphyromonas gulae* in plaque samples, 16S rRNA gene was amplified by polymerase chain reaction (PCR) using specific primers. All data were analyzed using Fisher’s exact probability test and odds ratio (OR) with a 95% confidence interval (95% CI). The prevalence of gingivitis was significantly higher in young cats (92.6%) than in young dogs (45.2%). The positive rate of spirochetes by PCR in gingivitis cases was 85.4% in young cats and 15.4% in young dogs, and the positive rate of *P*. *gulae* was 66.7% in young cats and 15.4% in young dogs. Both results were significantly higher in young cats than in young dogs. In young cats, spirochetes were significantly associated with gingivitis (OR = 7.95; 95% CI = 1.17, 53.83; P < 0.05), but *P*. *gulae* was not (OR = 2.44; 95% CI = 0.38, 15.66; P = 0.23). These results suggest that spirochetes may be associated with the early stages of periodontal disease in cats.

## Introduction

Gingivitis is a reversible inflammation confined to the gingiva, and it is described as an initial stage of periodontal disease [1]. Periodontal disease is a common disease in cats and dogs [2,3] and is thought to be initiated by oral bacteria in the plaque that adhere to the teeth. Recent studies have revealed a large diversity of bacterial species in the subgingival plaque of cats and dogs. There are also extensive differences between the microbiome identified in companion animals and humans [4–6]. Although healthy animals of the same species have similar composition of the oral microbiome, it changes with periodontal disease [7].

In dogs and cats, the incidence and severity of periodontal disease, especially periodontitis, are known to increase with age [8,9]. Conversely, although periodontitis is rarely seen in dogs or cats under one year old, gingivitis is a common diagnosis. Previous epidemiological studies showed that dogs aged between 0.5 and 1.5 years had no gingivitis [10] whereas approximately 50% of cats below one year of age have established gingivitis [11]. Thus, it is expected that the prevalence of gingivitis in young cats is higher than that in young dogs.

In humans, many bacterial species have been associated with the diagnosis of periodontal disease. Among them, *Treponema denticola*, a type of spirochete, belongs to the red complex species related to periodontal disease because of its virulence factors, along with *Tannerella forsythia* and *Porphyromonas gingivalis* [12,13]. Conversely, in dogs and cats, *Porphyromonas gulae* has been found to be the predominant species leading to periodontal disease [14–16], and research focusing on the role of spirochete is limited. However, recent reports have shown that the plaque from mild feline periodontitis contained a much larger number of *Treponema* species than those in dogs’ plaque [17,18].

We speculate that there may be an association between the high incidence of gingivitis in cats of an early age and the high incidence of spirochetes in cats with mild periodontal disease. Therefore, this study assessed the difference in the prevalence of gingivitis between young cats and dogs, clarifying the relationship between gingivitis and oral spirochetes in young cats.

## Materials and methods

### Subjects and ethical statement

Examination and sampling of the subjects were performed between April 2020 and March 2022 at Amica Pet Clinic, Yamaguchi, Japan. Cats and dogs younger than one year old were randomly examined. Cats and dogs were excluded from this study as samples if they had systemic underlying diseases that affect the oral cavity or oral diseases other than periodontal inflammatory diseases, or antibiotic treatment in the past month. Dental plaque was sampled and the oral cavity was checked, when the animals were anesthetized. Subgingival and supragingival samples were collected from all 99 animals.

Prospectively, the Yamaguchi University Ethical Committee on Animal Research counseled that because samples were obtained as a part of medical treatment, an Ethical Committee on Animal Research protocol was not required. All owners had given their written consent prior to participating in the study. No adverse events resulting from tissue acquisition were documented.

### Clinical evaluation of subjects

**Fig 1:**
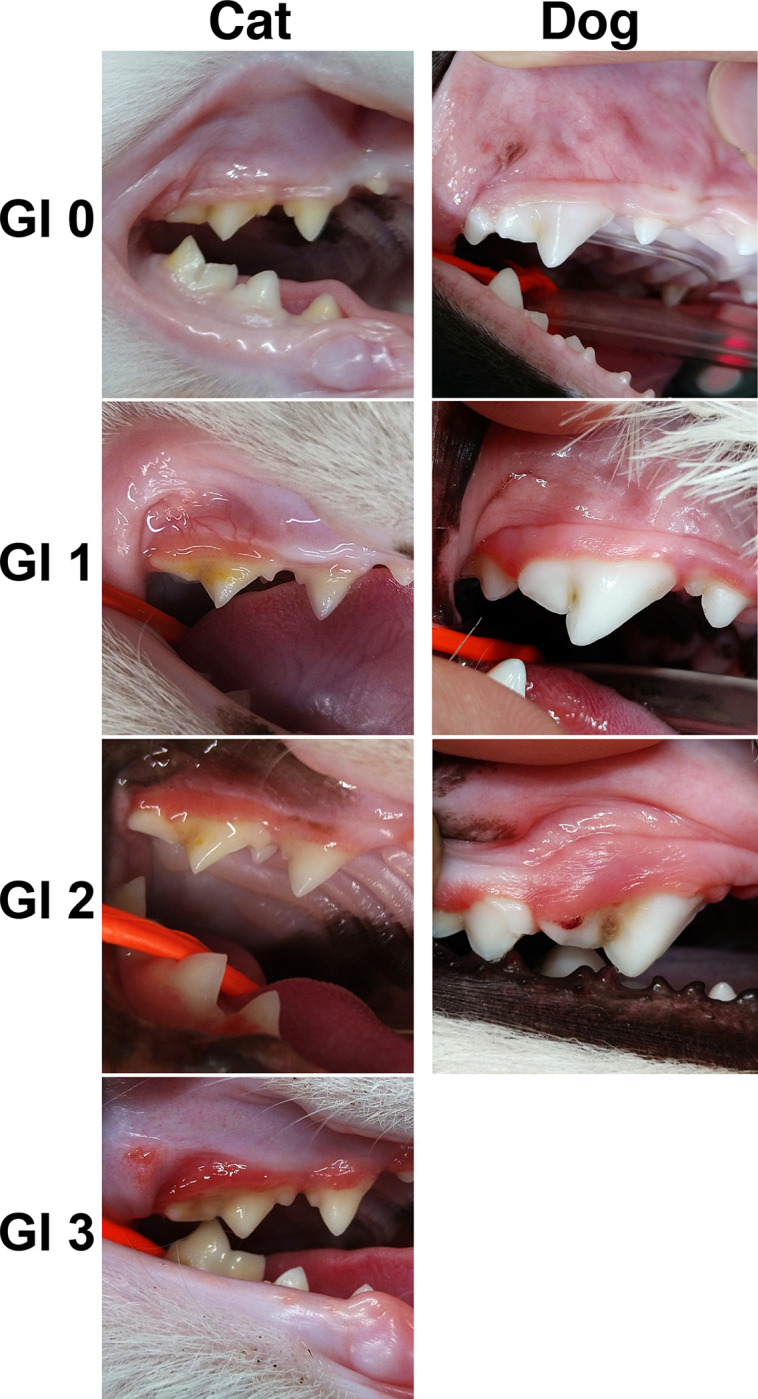
Examples of gingivitis determination according to the criteria for the GI systems in cats and dogs. The right maxillary fourth premolars (108) are compared.

Gingiva was evaluated based on the clinical characteristics of the Gingival Index (GI) system [19], classified in four levels from zero to three (Fig 1 and Table 1). The same evaluator determined the GI values for all samples. When determining only the presence or absence of gingivitis, it was regarded that GI 1–3 was positive and GI 0 was negative.

**Table 1:** Criteria for the Gingival Index (GI) systems.

GI Inflammation—Appearance	
0:	Absence of inflammation.
1:	Mild inflammation—slight change in color and little change in texture.
2:	Moderate inflammation—moderate glazing, redness, oedema, and hypertrophy. Bleeding on pressure.
3:	Severe inflammation—marked redness and hypertrophy. Tendency to spontaneous bleeding. Ulceration.

### Sample collection and preparation of examination

One site of the carnassials (maxillary fourth premolars or mandibular first molars) with the strongest gingival inflammation was targeted. A microbrush (TPC Disposable Micro Applicators Fine) was rubbed firmly five times on the gingival sulcus and the adjacent enamel of the buccal surface of the targeted tooth of each animal.

A part of the plaque sample attached to the microbrush was applied to a new, uncontaminated glass slide and stained with Hemacolor® (Merck, Germany) for microscopic examination. The remaining sample was placed in a tube containing 500 μL of phosphate-buffered saline (PBS) for DNA preparation. PBS samples were stored at −4°C until DNA extraction.

DNA was extracted and purified using a DNeasy Blood & Tissue Kit (QIAGEN, Netherlands) according to the manufacturer’s protocol for the polymerase chain reaction (PCR) method. DNA samples were stored at −30°C until needed.

### 16S ribosomal RNA gene PCR amplification

The specific sequence of the 16S ribosomal RNA gene was amplified using AGAGTTTGATCCTGGCTCAG and GTTACGACTTCACCCTCCT primers selective for the phylum Spirochaetes [20] or TTGCTTGGTTGCATGATCGG and GCTTATTCTTACGGTACATTCACA primers selective for *P*. *gulae* [14].

PCR was performed using GoTaq® Green Master Mix (Promega, USA) with the following cycling parameters: an initial denaturation at 95°C for 2 min, followed by 25 cycles of 30 s at 95°C, 30 s at 60°C, and 90 s at 72°C for the phylum Spirochaetes or 30 cycles of 30 s at 95°C, 30 s at 60°C, and 30 s at 72°C for *P*. *gulae*, with a final extension at 72°C for 5 min. The purity of the product was determined by electrophoresis in a 1% agarose gel using Mupid-2plus (Takara, Japan). DNA was stained with ethidium bromide and viewed under long-wavelength ultraviolet light using a UV transilluminator (ATTO, Japan).

### Statistical analysis

Data were analyzed using the software EZR 4.0.3 (Windows 11). Fisher’s exact test was used to compare the prevalence of gingivitis or positive rate of bacteria between cats and dogs, considering P < 0.05 as statistically significant. In addition, the association between the clinical characteristics of the gingiva and the detection of bacteria was evaluated by odds ratio (OR), with 95% confidence intervals (95% CI).

## Results

### Study population

A total of 68 cats (34 males and 34 females) and 31 dogs (14 males and 17 females) were sampled. The age of the cats varied between 5 and 12 months (mean, 7.1 months; SD, 1.4 months), and their weights ranged from 2.04 to 4.66 kg (mean, 3.22 kg; SD, 0.55 kg). The age of the dogs varied between 5 and 11 months (mean, 6.7 months; SD, 1.2 months), and their weights ranged from 1.44 to 27.0 kg (mean, 5.50 kg; SD, 3.34 kg). Further information, including sampling sites and GI of each animal, is shown in S1 and S2 Tables.

### Prevalence of gingivitis

**Fig 2:**
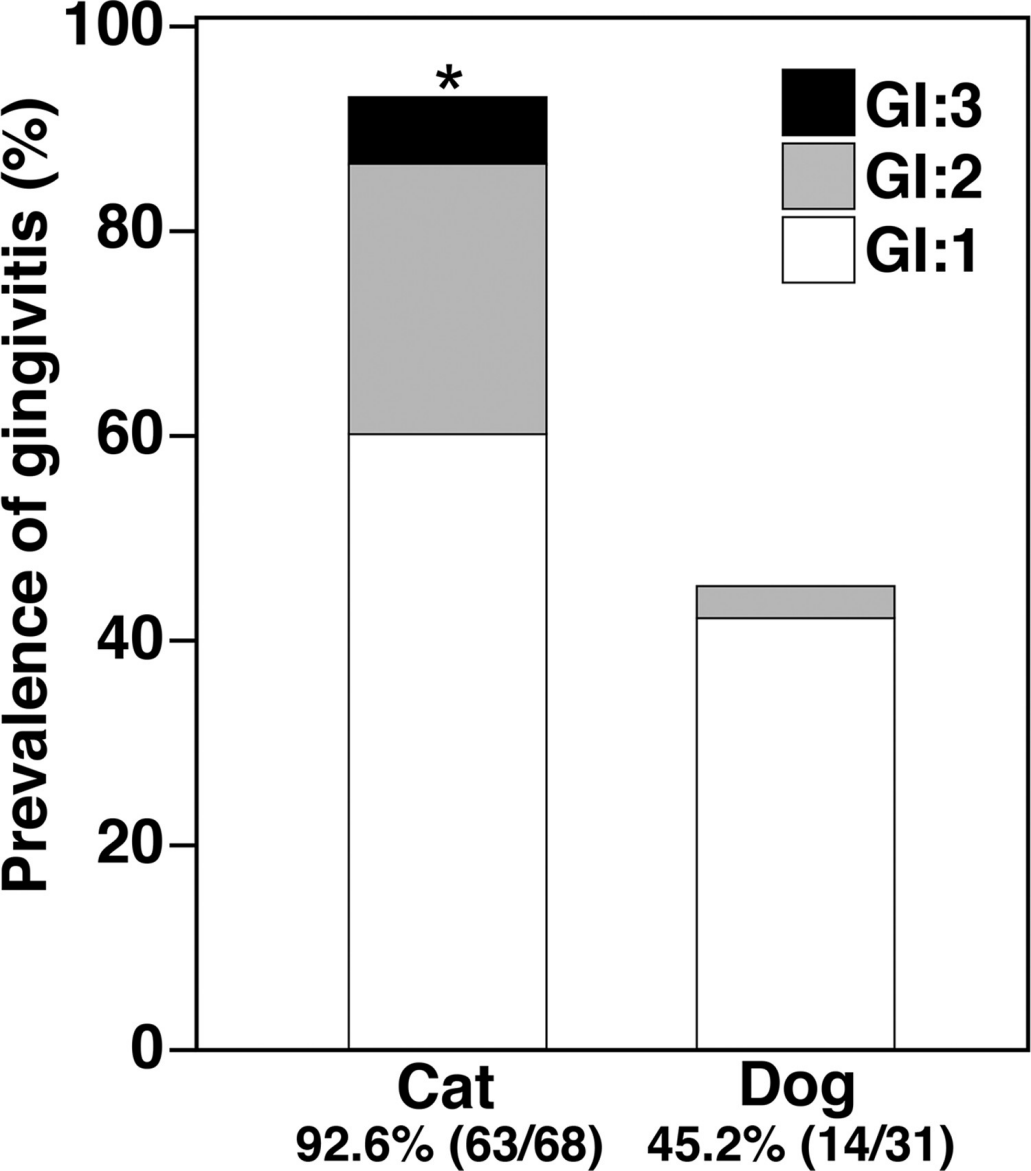
Prevalence of gingivitis in cats and dogs one year old or younger. Significant differences are indicated by asterisks (*P < 0.05).

The prevalence of gingivitis was 92.6% (63/68) in cats and 45.2% (14/31) in dogs (Fig 2). Cats had a significantly higher prevalence of gingivitis than dogs (P < 0.05).

### Microscopic examination

**Fig 3:**
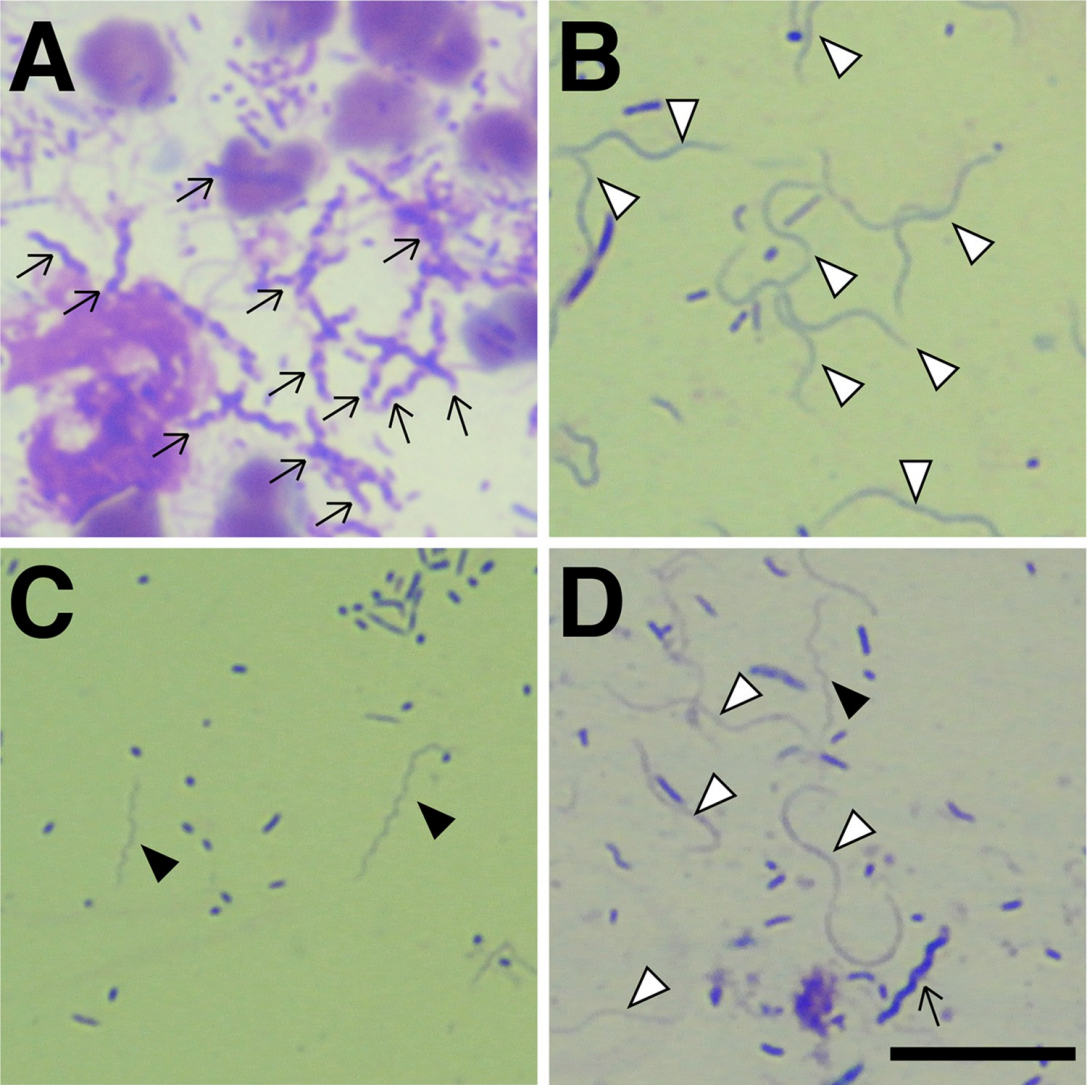
Several morphological types of spirochetes found in dental plaque. (A) High stainability and many spiral type (arrows). (B) Low stainability and little spiral type (white arrowheads). (C) Low stainability and many spiral type (black arrowheads). (D) Many types of spirochetes. Scale bar represents 10 μm.

To confirm if there were spirochetes in the dental plaque, the samples were stained and observed under an optical microscope. Several types of spirochetes were found based on their morphology (staining, spiral, and width) (Fig 3). At least one key morphological characteristic (low stainability, many spirals, and small width) was common to almost all spirochete-positive samples. The results were assessed by the presence or absence of spirochete (Table 2). Spirochetes were observed in samples of 51 of 63 cats (81.0%) and 2 of 14 dogs (14.3%) with gingivitis. Spirochetes were observed in samples from 2 of 5 cats (40.0%) and 1 of 17 dogs (5.9%) without gingivitis (Table 2).

**Table 2:** Rate of positivity for spirochetes and *P*. *gulae* by microscope or PCR examination.

		Microscope	PCR	
	N	Spirochetes (%)	Spirochetes (%)	*P*. *gulae* (%)
Cat	68			
with Gingivitis	63	51 (81.0)	53 (84.1)	39 (61.9)
without Gingivitis	5	2 (40.0)	2 (40.0)	2 (40.0)
Dog	31			
with Gingivitis	14	2 (14.3)	3 (21.4)	2 (14.3)
without Gingivitis	17	1 (5.9)	3 (17.3)	4 (23.5)

### Molecular analysis

The results of the PCR for the phylum Spirochaetes are shown in Table 2. A total of 53 of 63 (84.1%) samples from cats with gingivitis and 2 of 5 (40.0%) samples from cats without gingivitis were positive. A total of 3 of 14 (21.4%) samples from dogs with gingivitis and 3 of 17 (17.7%) samples from dogs without gingivitis were positive. Among animals with gingivitis, the detection rate of spirochete was significantly higher in cats than in dogs.

Only 1 of 53 microscopy positive cat samples and 1 of 3 microscopy positive dog samples were PCR negative and 3 of 15 microscopy negative cat samples and 4 of 28 microscopy negative dog samples were PCR positive for the phylum Spirochaetes (Table 3).

**Table 3:** The correlation between positive rates of spirochete by PCR or microscopic examination.

Cat		PCR (%)	
		+	−
Microscope (%)	+	52 (76.47)	1 (1.47)
	−	3 (4.41)	12 (17.65)
Dog		PCR (%)	
		+	−
Microscope (%)	+	2 (6.45)	1 (3.23)
	−	4 (12.90)	24 (77.42)

The PCR results for *P*. *gulae* are shown in Table 2. A total of 39 of 63 (61.9%) samples from cats with gingivitis and 2 of 5 (40.0%) samples from cats without gingivitis were PCR positive. A total of 2 of 14 (14.3%) samples from dogs with gingivitis and 4 of 17 (23.5%) samples from dogs without gingivitis were PCR positive. Among animals with gingivitis, the detection rate of *P*. *gulae* was significantly higher in cats than in dogs, as was the identification of spirochetes.

The association between the development of gingivitis in young cats and dogs and each bacterial species was examined by OR and 95% CI (Table 4). In young cats, spirochetes (OR = 7.95; 95% CI = 1.17, 53.83; P < 0.05) were shown to be significantly associated with gingivitis, but *P*. *gulae* (OR = 2.44; 95% CI = 0.38, 15.66; P = 0.23) was not associated with gingivitis. Conversely, in young dogs, neither spirochetes (OR = 1.27; 95% CI = 0.21, 7.58; P = 0.34) nor *P*. *gulae* (OR = 0.54; 95% CI = 0.08, 3.51; P = 0.29) was associated with gingivitis.

**Table 4:** Odds ratio of Spirochaetes or *P*. *gulae* for gingivitis.

	Odds Ratio	95% CI	*P* value
Cat			
The Phylum Spirochaetes	7.95	1.17–53.83	<0.05
*P*. *gulae*	2.44	0.38–15.66	0.23
Dog			
The Phylum Spirochaetes	1.27	0.21–7.58	0.34
*P*. *gulae*	0.54	0.08–3.51	0.29

## Discussion

To the best of our knowledge, this study reported for the first time a higher prevalence of gingivitis in young cats under one year old by comparing to young dogs. Because gingivitis in small animals presents with few clinical signs, it can only be diagnosed as a pathology with careful observation within the oral cavity. The fact that many veterinarians and owners are unaware of the signs of gingivitis in young animals may have contributed to underdiagnosing this disease.

Periodontal disease is the most common and important health problem in cats and dogs. Gingivitis is the initial, reversible, and preventable stage of periodontal disease, and it may develop to periodontitis, involving the progressive and irreversible destruction of the periodontal tissues [1,21]. Complications include chronic ulcerative paradental stomatitis, faucitis, and chronic gingivostomatitis [22]. Therefore, it is desirable to swiftly diagnose gingivitis cases. The 2019 AAHA Dental Care Guidelines for Dogs and Cats [21] recommends that a true dental prophylaxis starts at one year of age for cats and small- to medium-breed dogs and by two years of age for larger-breed dogs, even if there are no obvious lesions. The clarification of the association between gingivitis in young cats and oral bacteria should help in establishing effective prevention and providing treatment.

Various forms of spirillum have been confirmed in the plaque of dogs and cats at the stage of preliminary experiments, in addition to *Treponema*, which is usually detected in the oral cavity of humans. Nonetheless, other spirochetal species (such as *Borrelia* and *Brachyspira*) may coexist. Therefore, in this study, primers specific to the phylum Spirochaetes were used to detect spirochetes. The results found by PCR were well correlated with the pictures obtained by microscopy. Because one similar morphology was common to almost all spirochete-positive samples under light microscopy, PCR-positive results were considered to reflect the presence of a particular species.

In one sample, spirochetes were detected under a microscope but not by PCR for both cats and dogs. This may be due to both samples presenting a small number of spirochetes on the smear; the number of bacteria in the sample may be below the detection limit for PCR. It was also possible that there were novel spirochetes that did not match the primer pair used [23] or that an inhibitor was present in the sample.

It is reported that that the microbiota of periodontally healthy cats were distinguishable from diseased cats [24]. In this study, we showed that spirochetes are more associated with gingivitis in young cats than with *P*. *gulae*. *P*. *gulae* is thought to be associated with periodontal disease in cats, and a correlation has been reported between the proportion of this species and the severity of periodontal disease [16]. However, because the subjects of the study were the animals with gingivitis (mild periodontal disease), the association of *P*. *gulae* to the disease may not be significant.

Many studies reported that spirochetes as a group and *T*. *denticola* are associated with periodontal disease, especially periodontitis in humans [25]. One of the reports showed that *T*. *denticola* increases susceptibility to gingivitis [26]. On the other hand, only a few studies have investigated the association of oral spirochetes with periodontal disease in animals. However, in recent years, some studies of the subgingival microbiota using next-generation sequencing have found spirochetes to have a higher abundance in periodontally diseased cats compared to healthy [18,24]. Moreover, if there might be more diverse oral spirochetes in dogs and cats than in humans as previous report have shown [23], it is also possible that there are spirochetes with etiologies that are not common in humans. The association of spirochetes with gingivitis in young cats shown in this study suggests that spirochetes, together with other oral microbes or alone, may play some role in the early stages of periodontal disease in cats.

## Supporting information

S1 Table Cat samples used in this study. (DOCX)

S2 Table Dog samples used in this study. (DOCX)
