# Supplementary material for: The association between gingivitis and oral spirochetes in young cats and dogs
Source: PLoS One. 2023 Jan 27;18(1):e0281126. doi: 10.1371/journal.pone.0281126 (PMC9882964; doi:10.1371/journal.pone.0281126)
Supplement: S1 Table — (DOCX) [file pone.0281126.s001.docx]

| No. | Age (months) | Breed | Sex | Weight (kg) | Sampling teeth site | GI | Spirochete (microscope) | Spirochaetes (PCR) | *P. gulae* (PCR) |
| --- | --- | --- | --- | --- | --- | --- | --- | --- | --- |
| 1 | 12 | Domestic Shorthair | FS | 4.66 | 108 | 1 | + | + | + |
| 2 | 6 | Domestic Shorthair | M | 2.65 | 208 | 1 | + | + | + |
| 3 | 12 | Exotic Shorthair | M | 3.62 | 208 | 1 | + | + | + |
| 4 | 6 | Munchkin | F | 2.54 | 108 | 2 | + | + | + |
| 5 | 11 | Domestic Shorthair | M | 3.86 | 108 | 1 | + | + | － |
| 6 | 8 | Domestic Shorthair | M | 4.2 | 108 | 1 | － | － | + |
| 7 | 7 | Siberian | F | 3.4 | 108 | 1 | － | － | + |
| 8 | 6 | Domestic Shorthair | M | 3.2 | 108 | 3 | + | + | + |
| 9 | 7 | Munchkin | F | 3.02 | 108 | 2 | + | + | + |
| 10 | 9 | Domestic Shorthair | F | 2.42 | 108 | 1 | + | + | － |
| 11 | 6 | Munchkin | F | 2.56 | 108 | 0 | － | － | － |
| 12 | 6 | Munchkin | F | 2.36 | 108 | 0 | － | － | － |
| 13 | 6 | Mixed Breed | M | 4.08 | 208 | 2 | － | + | － |
| 14 | 6 | Somali | F | 2.2 | 108 | 2 | + | + | + |
| 15 | 8 | Scottish Fold | M | 3.98 | 208 | 1 | － | － | － |
| 16 | 6 | Domestic Shorthair | M | 4.14 | 108 | 2 | + | + | + |
| 17 | 5 | Domestic Shorthair | M | 3.08 | 208 | 1 | + | + | + |
| 18 | 6 | Munchkin | M | 3.22 | 208 | 1 | － | － | － |
| 19 | 12 | Domestic Shorthair | M | 4.52 | 108 | 1 | + | + | + |
| 20 | 6 | Domestic Shorthair | M | 3.52 | 108 | 1 | + | + | + |
| 21 | 6 | Domestic Shorthair | F | 3.06 | 208 | 2 | － | － | － |
| 22 | 6 | Domestic Shorthair | M | 2.82 | 108 | 3 | + | + | + |
| 23 | 6 | Domestic Shorthair | F | 2.5 | 208 | 1 | + | + | + |
| 24 | 6 | Domestic Shorthair | M | 2.22 | 108 | 2 | + | + | + |
| 25 | 6 | Domestic Shorthair | M | 2.9 | 108 | 1 | + | + | + |
| 26 | 10 | Scottish Fold | F | 3.58 | 208 | 1 | + | + | + |
| 27 | 5 | Domestic Shorthair | F | 2.9 | 208 | 1 | + | + | + |
| 28 | 6 | Domestic Shorthair | F | 2.6 | 108 | 1 | + | + | － |
| 29 | 6 | Domestic Shorthair | F | 2.7 | 208 | 1 | + | + | － |
| 30 | 6 | Munchkin | F | 2.04 | 208 | 2 | + | + | － |
| 31 | 10 | Domestic Shorthair | M | 3.85 | 108 | 1 | + | + | + |
| 32 | 7 | Domestic Shorthair | F | 2.66 | 208 | 1 | + | + | + |
| 33 | 7 | Domestic Shorthair | M | 4.6 | 108 | 1 | － | － | － |
| 34 | 6 | Domestic Shorthair | M | 2.8 | 108 | 1 | + | + | + |
| 35 | 7 | Domestic Shorthair | M | 3.9 | 208 | 2 | + | + | + |
| 36 | 6 | Domestic Shorthair | F | 2.34 | 108 | 1 | － | + | － |
| 37 | 6 | Domestic Shorthair | F | 3.04 | 208 | 1 | － | + | + |
| 38 | 7 | Domestic Shorthair | F | 2.5 | 108 | 2 | + | + | + |
| 39 | 6 | Domestic Shorthair | F | 2.96 | 108 | 2 | + | + | + |
| 40 | 6 | Domestic Shorthair | M | 3.2 | 108 | 0 | + | + | + |
| 41 | 6 | Domestic Shorthair | M | 3.2 | 208 | 0 | + | + | + |
| 42 | 6 | Domestic Shorthair | F | 2.66 | 108 | 1 | + | + | － |
| 43 | 7 | Domestic Shorthair | M | 4.22 | 108 | 1 | + | + | + |
| 44 | 10 | Domestic Shorthair | M | 3.36 | 108 | 3 | + | + | + |
| 45 | 10 | Domestic Shorthair | F | 3.48 | 108 | 1 | + | + | － |
| 46 | 7 | Domestic Shorthair | M | 3.96 | 108 | 1 | + | + | － |
| 47 | 10 | Munchkin | F | 2.94 | 108 | 1 | － | － | － |
| 48 | 6 | Domestic Shorthair | F | 2.38 | 108 | 1 | + | + | + |
| 49 | 7 | Domestic Shorthair | F | 3.34 | 108 | 1 | + | + | － |
| 50 | 10 | Domestic Shorthair | F | 2.6 | 108 | 2 | + | + | + |
| 51 | 11 | Domestic Shorthair | F | 3.62 | 108 | 1 | + | + | + |
| 52 | 12 | Domestic Shorthair | F | 3.58 | 108 | 1 | － | － | － |
| 53 | 7 | Domestic Shorthair | F | 3.1 | 208 | 2 | + | + | － |
| 54 | 6 | Domestic Shorthair | M | 3.44 | 108 | 2 | + | + | + |
| 55 | 6 | Munchkin | M | 2.9 | 208 | 1 | + | + | + |
| 56 | 6 | Domestic Shorthair | F | 2.24 | 108 | 3 | + | + | + |
| 57 | 7 | Scottish Fold | M | 3.82 | 108 | 1 | － | － | － |
| 58 | 6 | Domestic Shorthair | M | 3.7 | 108 | 1 | + | + | + |
| 59 | 6 | Domestic Shorthair | M | 3.12 | 108 | 2 | + | + | － |
| 60 | 6 | Domestic Shorthair | F | 2.84 | 108 | 2 | + | + | － |
| 61 | 6 | Domestic Shorthair | M | 3.56 | 108 | 2 | + | － | － |
| 62 | 6 | Domestic Shorthair | F | 3.1 | 108 | 1 | + | + | － |
| 63 | 6 | Domestic Shorthair | M | 4.15 | 108 | 2 | + | + | － |
| 64 | 6 | Domestic Shorthair | M | 4.12 | 108 | 1 | + | + | + |
| 65 | 6 | Domestic Shorthair | M | 4.58 | 108 | 0 | － | － | － |
| 66 | 6 | Domestic Shorthair | M | 3.4 | 108 | 1 | + | + | + |
| 67 | 6 | Domestic Shorthair | F | 2.78 | 108 | 1 | + | + | + |
| 68 | 7 | Domestic Shorthair | F | 2.54 | 108 | 1 | + | + | + |

S1_table. Cat samples used in this study.
