# Supplementary material for: The association between gingivitis and oral spirochetes in young cats and dogs
Source: PLoS One. 2023 Jan 27;18(1):e0281126. doi: 10.1371/journal.pone.0281126 (PMC9882964; doi:10.1371/journal.pone.0281126)
Supplement: S2 Table — (DOCX) [file pone.0281126.s002.docx]

| No. | Age (months) | Breed | Sex | Weight (kg) | Sampling teeth site | GI | Spirochete (microscope) | Spirochaetes (PCR) | *P. gulae* (PCR) |
| --- | --- | --- | --- | --- | --- | --- | --- | --- | --- |
| 1 | 8 | Miniature Dachshund | F | 2.8 | 208 | 0 | + | + | + |
| 2 | 9 | Shiba | M | 7.2 | 208 | 0 | － | + | + |
| 3 | 7 | Shiba | F | 8.1 | 208 | 0 | － | － | － |
| 4 | 6 | Mongrel | M | 14.85 | 108 | 0 | － | + | － |
| 5 | 6 | Miniature Dachshund | F | 3.4 | 108 | 0 | － | － | + |
| 6 | 5 | Shiba | M | 7.46 | 108 | 0 | － | － | － |
| 7 | 6 | Toy Poodle | F | 3.14 | 108 | 0 | － | － | － |
| 8 | 6 | Chihuahua | F | 2.4 | 108 | 1 | － | － | － |
| 9 | 5 | Mixed Breed | M | 2.64 | 208 | 0 | － | － | － |
| 10 | 7 | Pembroke Welsh Corgi | M | 12.8 | 409 | 1 | + | + | － |
| 11 | 7 | Chihuahua | F | 2.3 | 208 | 1 | － | － | － |
| 12 | 11 | Mongrel | M | 4.12 | 108 | 1 | － | － | － |
| 13 | 8 | Chihuahua | F | 2.66 | 208 | 0 | － | － | － |
| 14 | 6 | Miniature Dachshund | F | 3.88 | 409 | 1 | － | － | － |
| 15 | 11 | Miniature Dachshund | M | 5 | 309 | 1 | － | － | － |
| 16 | 6 | Chihuahua | M | 2.26 | 108 | 1 | － | － | + |
| 17 | 8 | Golden Retriever | M | 27 | 108 | 1 | － | － | － |
| 18 | 6 | Chihuahua | M | 1.44 | 208 | 1 | － | + | － |
| 19 | 6 | Toy Poodle | F | 1.8 | 108 | 0 | － | － | － |
| 20 | 6 | Pug | F | 5.82 | 108 | 0 | － | － | + |
| 21 | 6 | Pomeranian | M | 2.7 | 108 | 0 | － | － | － |
| 22 | 6 | Miniature Dachshund | F | 3.88 | 108 | 0 | － | － | － |
| 23 | 6 | Toy Poodle | M | 2 | 409 | 1 | － | － | － |
| 24 | 5 | Pomeranian | F | 3.6 | 108 | 0 | － | － | － |
| 25 | 8 | Miniature Dachshund | M | 5.7 | 108 | 0 | － | － | － |
| 26 | 5 | Mongrel | M | 12.26 | 108 | 2 | + | － | － |
| 27 | 8 | Toy Poodle | F | 3.85 | 108 | 1 | － | － | － |
| 28 | 6 | Toy Poodle | F | 3.4 | 108 | 0 | － | － | － |
| 29 | 6 | Miniature Dachshund | F | 4.14 | 409 | 1 | － | + | + |
| 30 | 6 | Italian Greyhound | F | 4.58 | 108 | 0 | － | － | － |
| 31 | 7 | Toy Poodle | F | 3.2 | 108 | 1 | － | － | － |

S2_table. Dog samples used in this study.
